# Very small embryonic/epiblast-like stem cells (VSELs) and their potential role in aging and organ rejuvenation – an update and comparison to other primitive small stem cells isolated from adult tissues

**DOI:** 10.18632/aging.100449

**Published:** 2012-04-07

**Authors:** Mariusz Z. Ratajczak, Dong-Myung Shin, Rui Liu, Kasia Mierzejewska, Janina Ratajczak, Magda Kucia, Ewa K. Zuba-Surma

**Affiliations:** ^1^ Stem Cell Biology Program at the James Graham Brown Cancer Center, University of Louisville, Louisville, KT, USA; ^2^ Department of Physiology Pomeranian Medical University, Szczecin, Poland; ^3^ Department of Cell Biology, Faculty of Biochemistry, Biophysics and Biotechnology, Jagiellonian University, Krakow, Poland

**Keywords:** VSELs, Igf2-H19, RasGRF1, imprinted genes

## Abstract

Very small embryonic-like stem cells (VSELs) are a population of developmentally early stem cells residing in adult tissues. These rare cells, which are slightly smaller than red blood cells, i) become mobilized during stress situations into peripheral blood, ii) are enriched in the Sca1^+^Lin^−^CD45^−^ cell fraction in mice and the CD133^+^ Lin^−^CD45^−^ cell fraction in humans, iii) express markers of pluripotent stem cells (e.g., Oct4, Nanog, and SSEA), and iv) display a distinct morphology characterized by a high nuclear/cytoplasmic ratio and undifferentiated chromatin. Recent evidence indicates that murine VSELs are kept quiescent in adult tissues and protected from teratoma formation by epigenetic modification of imprinted genes that regulate insulin/insulin like growth factor signaling (IIS). The successful reversal of these epigenetic changes in VSELs that render them quiescent will be crucial for efficient expansion of these cells. The most recent data *in vivo* from our and other laboratories demonstrated that both murine and human VSELs exhibit some characteristics of long-term repopulating hematopoietic stem cells (LT-HSCs), are at the top of the hierarchy in the mesenchymal lineage, and may differentiate into organ-specific cells (e.g., cardiomyocytes). Moreover, as recently demonstrated the number of these cells positively correlates in several murine models with longevity. Finally, while murine BM-derived VSELs have been extensively characterized more work is needed to better characterize these small cells at the molecular level in humans.

## INTRODUCTION

One of the most intriguing questions in stem cell biology is whether pluripotent stem cells (PSCs) exist in adult tissues. Several groups of investigators employing i) various isolation protocols, ii) detection of surface markers, and iii) experimental *in vitro* and *in vivo* models, have reported the presence of cells that possess a pluripotent character in adult tissues [1-4]. Such cells were assigned various operational abbreviations and names in the literature that added confusion to the field and raised the basic question of whether these are truly distinct or overlapping populations of the same primitive stem cells. Unfortunately, these cells were never characterized side-by-side to address this important issue. Nevertheless, taking into consideration their common features described in the literature, it is very likely that various investigators have described overlapping populations of developmentally early stem cells that are closely related [1-6].

From a developmental point of view, another important question is why should PSCs reside in adult organs? For many years it has been accepted that adult tissues contain only tissue-committed stem cells (TCSCs), such as epidermal stem cells, hematopoietic stem cells, or skeletal muscle stem cells, that have a limited potential for differentiation [7-11]. To address this question, we consider two scenarios that could occur during early embryogenesis and the development of lineage-restricted TCSCs [1,12,13]. In the first scenario, PSCs present in the inner cell mass of the blastocyst/epiblast, after giving rise to more differentiated lineage-restricted TCSCs, gradually disappear from the growing embryo and do not reside in adult tissues. In the second scenario, which we believe is more likely to take place during embryogenesis, some PSCs give rise to TCSCs but some survive in adult tissues as a backup population of PSCs that renews the pool of TCSCs over time. In this scenario, PSCs are precursors of TCSCs during organ/tissue rejuvenation and a source of these cells in emergency situations when organs are damaged (e.g., heart infarct or stroke). This scenario, however, requires such PSCs population deposited in adult tissues to be kept under control and in a quiescent state, which is essential to preventing uncontrolled proliferation leading to teratoma formation.

In this review, we will discuss the second scenario, in which population of very small embryonic- like stem cells (VSELs) - a rare population of epiblast-derived PSCs deposited in adult tissues is a reserve pool for TCSCs [1,14,15]. We will also present the most recent observations from our and other laboratories that support the presence of developmentally primitive stem cells in adult tissues with broad potential to differentiate into multiple lineages, which correspond to the VSELs described by our team.

### Data supporting the presence of small stem cells in adult tissues

As mentioned above, several primitive cells with the characteristics of pluripotent or multipotent stem cells were isolated from adult murine and human tissues after expansion in *in vitro* cultures of cell suspensions isolated from murine and human bone marrow (BM) or human umbilical cord blood (UCB) or derived from adult organs after enzymatic processing by proteolytic enzymes [3-5,16,17]. In these *in vitro* cultures, in which cells grow while adhering to plastic or fibronectin, several populations of primitive cells were isolated, expanded and assigned different operational names [2,3,5,6,17-20]. Unfortunately, in none of these isolation procedures the phenotype of stem cell that initiated these cultures has been described clear from the beginning and the expanded in vitro cells were variously described as multipotent adult stem cells (MASCs) [19], unrestricted somatic stem cells (USSCs) [4,21,22] or marrow-isolated adult multilineage-inducible (MIAMI) cells [2].

In parallel, other isolation strategies were also employed, and an interesting population of small cells (ELH stem cells) able to differentiate into epithelial cells and hematopoietic cells was isolated from the murine BM by elutriation (E), lineage depletion (L), and the ability to home (H) to BM [23-25]. On the other hand, small cells able to differentiate into cells from all germ layers and called “spore-like stem cells” were isolated from adult mammalian tissues; however, the strategy by which they were purified and which surface markers they express was not provided in the original report [26].

Nevertheless, the presence of these primarily non-hematopoietic stem cells residing in adult BM challenged the concept of stem cell plasticity, which was supported by some data suggesting that hematopoietic stem cells (HSCs) may trans-differentiate into cells for non-hematopoietic tissues in murine models of experimental heart infarct, stroke, or liver damage [27,28]. The data in some of the experiments that showed a contribution of grafted BM cells into non-hematopoietic lineages gave the false impression that hematopoietic stem cells (HSCs) had trans-differentiated into cells for non-hematopoietic tissues [29,30].

Rejecting the concept that trans-differentiation of adult HSCs into non-hematopoietic lineages is a frequent biological phenomenon, our main aim became to isolate a population of true PSCs from BM, which could explain the data showing a contribution from BM cells in the regeneration of damaged organs. Based on preliminary data gained from our initial experiments, we purified from adult murine BM a population of cells slightly smaller than red blood cells that expressed the surface phenotype Sca1^+^Lin^−^CD45^−^ and markers of pluripotent stem cells (*e.g*., Oct4, Nanog, and SSEA-4). These cells displayed a distinct morphology characterized by a high nuclear/cytoplasmic ratio and undifferentiated chromatin [31, 32]. Phenotypically similar cells were subsequently purified from murine fetal liver, as well as from several adult murine organs, including brain, skeletal muscle, kidney, lung, liver, and pancreas [33-36].

Small cells comprising population of human VSELs were also purified from neonatal umbilical cord blood (UCB) and adult patient-mobilized peripheral blood (mPB) [37-43]. Human UCB and mPB are enriched in VSELs within a population of CD133^+^Lin^−^CD45^−^ cells that co-express CXCR4, and some of them are also CD34^+^. Like their murine counterparts, human UCB and mPB VSELs also express Oct4 and Nanog transcription factors in nuclei and express SSEA-1 on the surface [37-42]. The procedure for how to purify these cells has been described in detail in several publications and summarized in *Current Protocols in Cytometry* [44] and *Methods in Cell Biology* [14]. In this review, we will focus on new observations generated by our team, as well as the work of other groups working on VSELs or closely related cells.

### Reports by other groups on cells that are attributable to VSELs and closely related cells

The most common feature of VSELs is that they possess very primitive morphology and relatively small size [31,32,37,39]. Recently, there have been several published reports that support the existence of small, primitive VSELs or VSEL-like cells in adult tissues, and we will briefly discuss the most important of these observations [45-51].

In one report, a population of murine VSELs was isolated from BM, and in a set of elegant experiments, these cells were demonstrated to be able to give rise to mesenchymal and endothelial lineages [49]. The bone-forming activity of these cells, if embedded in gelatin sponges and implanted into living mice, exceeded the activity of other populations of BM-purified cells tested in the same assay. Based on this finding, it has been proposed that Sca-1^+^Lin^−^CD45^−^ VSELs are at the top of the hierarchy for the mesenchymal and endothelial lineages in BM [49].

In another report, VSELs were purified from rat BM and successfully employed to regenerate damaged myocardium in an experimental rodent model of acute myocardial infarction [52]. These cells expressed SSEA-1 antigen on their surface and Oct-4 in their nuclei. Similarly, small non-hematopoietic murine BM-derived cells that correspond in size to VSELs were also shown to give rise to type 2 pneumocytes, which produce lung surfactant protein after transplantation into surfactant-deficient mice [46]. In order to determine whether surfactant-producing epithelial cells were derived from the non-hematopoietic or the hemato-poietic fraction of BM cells that were used to treat surfactant-deficient mice, the authors employed a lineage-tracing approach in which Cre recombinase-specific vav-promoter-activated green fluorescence protein (GFP) from a ROSA-GFP was used as a reporter transgene [46]. Small cells with VSEL markers were also identified in murine neonatal retina [36], and in another report it was demonstrated that small non-hematopoietic lineage-negative (lin^−^) cells isolated from adult BM by elutriation (Fraction 25 or Fr25) were involved in retinal regeneration following the induction of anterior ischemic optic neuropathy and optic nerve crush in a rodent model [53]. Similar population of non-hematopoietic CD45-negative small stem cells harvested from BM via elutriation, has been recently shown to give rise into functional insulin-producing cells *in vivo* in chemically induced diabetic mice [45]. Finally, several features of VSELs are displayed in so-called multilineage-differentiating, stress-enduring (Muse) cells recently isolated from murine and human BM populations [48,54]. These cells may play a major role as populations of cells that preferentially give rise to induced pluripotent stem cells (iPSCs) when BM-derived stromal cells are induced to pluripotency by genetic manipulation [48,54].

The presence of VSELs in human tissues has also been supported by several recent publications. In particular, UCB-VSELs have been successfully purified by another team [55], and cells corresponding to VSELs were purified from UCB by other investigators and described as a population of UCB-derived embryonic-like stem cells [17,56,57]. A corresponding population of primitive stem cells that resembles VSELs named as Omnicytes was also described as circulating in UCB and being capable to migrate into maternal circulatory system [58,59]. Finally, cells similar to UCB-VSELs were reported to reside in an ovarian epithelial layer in postmenopausal ovaries [47].

Thus, mounting evidence supports the conclusion that small cells described as VSELs or VSEL-like cells that express developmentally primitive markers reside in adult murine and human tissues. However, to confirm that these populations of small primitive stem cells, such as “Muse” stem cells [48, 54], small non-hematopoietic Fr25/Lin^−^ BM-derived stem cells [53], or ELH stem cells [23, 24], overlap with VSELs requires further direct comparison. The detailed protocols on how to purify VSELs are published and we would be happy to share with other groups our expertise in how to isolate these rare cells. Most importantly, VSELs are currently purified in several laboratories worldwide and the coming years will bring more information on their biology and *in vitro* and *in vivo* differentiation potential.

### Recent observations from our group on murine and human cells that are attributable to VSELs

In the last years we have put considerable effort into better characterizing these rare cells at the molecular level.

#### - Progress on VSEL isolation strategies

VSELs from human UCB and mPB were initially purified from an erythrocyte-depleted population of nucleated cells by multiparameter sorting as a population of small CD133^+^ CD45^−^ Lin^−^ cells [37]. However, this procedure is time consuming and the sorting time required to process an entire cord blood unit (~50-100 ml) to isolate rare VSELs from UCB mononucleated cells takes up to 3-4 days [37]. Therefore, from the beginning it has been clear that a more rapid and less-expensive method for isolation of these cells must be found. In order to establish a more efficient method for VSEL purification from UCB, we employed a three-step isolation strategy based on removal of erythrocytes by hypotonic lysis (1^st^ step), followed by immunomagnetic separation of CD133^+^ cells (2^nd^ step), followed by FACS-based isolation of CD133^+^Lin^−^CD45^−^ cells (3^rd^ step). The entire isolation procedure takes only 2-4 hours per 100 ml of UCB [39,44].

As an alternative strategy, we recently proposed to reduce the number of lineage factors required for purification by exposure of erythrocyte-depleted, immunomagnetic bead-selected CD133^+^ cells to Aldefluor, followed by staining with anti-CD133^+^ antibodies and only two lineage-specific antibodies (anti-CD45 and anti-GlyA) for step 3 [38]. Aldefluor is a substrate for aldehyde-dehydrogenase (ALDH), a cytosolic enzyme highly expressed in less-differentiated hematopoietic cells, and is implicated in resistance to some alkylating agents [60,61]. In the presence of ALDH, Aldefluor becomes modified to a fluorescent molecule that may then be used to mark ALDH-expressing cells. Inclusion of anti-GlyA antibody is based on the well-known fact that small erythroblast GlyA^+^ cells present in UCB do not express the CD45 antigen.Thus, selection for CD45^−^ cells could enrich for these unwanted cells. By employing this strategy, we are able to subsequently sort CD133^+^ cells enriched for VSELs and are able to obtain ~10^3^ CD133^+^/ CD45^−^/GlyA^−^/ALDH^low^and 4x10^3^ CD133^+^/ CD45^−^/GlyA^−^/ALDH^high^ UCB-VSELs from 100 ml of UCB [38]. These numbers demonstrate how rare these cells are in UCB. When we compared both fractions of VSELs by RT-PCR gene expression analysis, we found that CD133^+^/CD45^−^/GlyA^−^/ALDH^low^VSELshave a higher expression of *Oct4* than the CD133^+^/CD45^−^/GlyA^−^/ALDH^high^ fraction [38].

However, we are aware that there is yet room to improve sorting and to develop alternative strategies for purifying UCB-VSELs. To accomplish this goal, one should consider the use of metabolic fluorochromes to see whether VSELs are enriched among Rh123^dull^, Pyronin Y^low^, and Hoechst 33342^low^ cells. Moreover, we expect that our proteomics data analysis with UCB-VSELs will reveal the presence of new positive markers that could be employed for purification of these rare cells.

#### - Mobilization of VSELs into PB in response to organ/tissue damage

VSELs circulate in PB under steady-state conditions; however, the number of these cells is very low. In our recent work we provided evidence that VSELs can be mobilized into peripheral blood in adult patients injected with granulocyte-colony stimulating factor (G-CSF). This observation laid the foundation for the concept that G-CSF mobilization can be employed to harvest these cells from patients for therapeutic purposes.

Furthermore, our studies on VSEL mobilization into PB revealed that VSELs are mobilized not only, as described previously, in patients suffering from heart infarct [40,42,62] or stroke [41] but also in patients suffering from skin burns [63], active inflammatory bowel disease [64], and cancer [14]. More importantly, based on our preliminary data, we also envision that the number of VSELs circulating in PB in patients could be of prognostic value. This however, requires further study and long-term clinical correlations.

#### - Novel characteristics of human VSELs

In the past two years we investigated several experimental approaches to better characterizing murine and human UCB-derived VSELs, both at the molecular and morphological levels. First, our molecular gene array analysis studies on limited numbers of highly purified VSELs revealed that murine VSELs are somewhat heterogenous developmentally [65-68]. In particular, while all of them express the pluripotency marker Oct-4, some of them express genes that are more closely related to genes expressed by epiblast-derived stem cells and others to genes expressed by migrating primordial germ cells. This shows that VSELs resemble early stem cells developmentally; if isolated from adult BM, they may differ somewhat in their differentiation potential [65-68].

**Figure 1:**
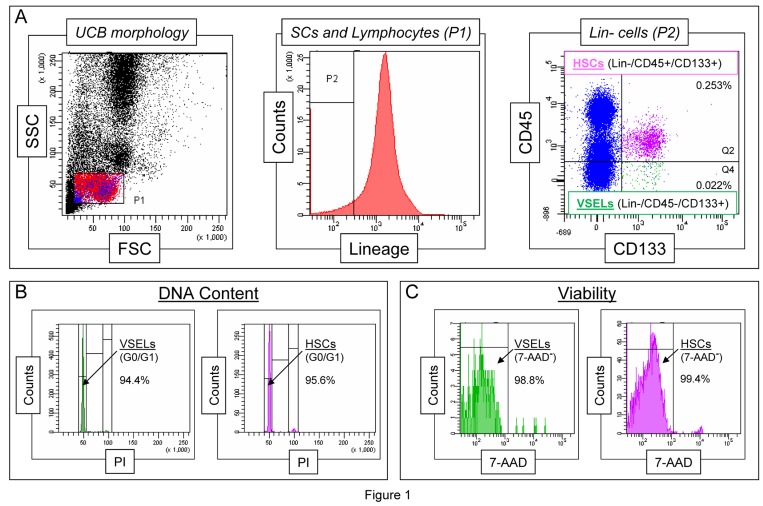
Morphological characteristics of UCB-derived VSELs by flow cytometric assays **Panel A** shows a well-established flow cytometric protocol for identification of CD133^+^Lin^−^CD45^−^ VSELs among the whole-nucleated fraction of UCB cells. The dot-plot (left) visualizes cells based on FSC and SSC parameters, indicating the relative cellular size and complexity, respectively. The FSC^low^/SSC^low^ lymphocytic population, which includes very small objects, is enclosed in region P1, and the cells were further analyzed according to hematopoietic lineage marker expression (middle histogram). Lin- cells from region P2 are plotted in the dot-plot representing CD133 and CD45 expression (right). VSELs are identified as CD133+Lin^−^CD45^−^ cells (region Q4), while HSCs are identified as CD133^+^Lin-CD45^+^ cells (region Q2). Percentages indicate the content of both stem cell populations among all nucleated cells in one representative UCB sample. **Panel B** presents a representative analysis of DNA content in UCB-derived VSELs and HSCs following fixation and staining with propidium iodide (PI). The percentages indicate normal diploid (2n) VSEL and HSC fractions in the G0/G1 phase of the cell cycle. **Panel C** shows the viability of VSELs and HSCs examined by flow cytometry following the staining of freshly isolated cells with a viability dye, 7-aminoactinomycin D (7-AAD). Viable VSELs and HSCs are represented as 7-AAD^−^ cells, which exclude the dye, and their percentage content is computed for each of the stem cell populations.

Based on well-established FACS identification protocols, we also found that CD133^+^Lin^−^CD45^−^VSELs identified in UCB (Figure 1 panel A), like their murine counterparts, i) highly express telomerase (not published), ii) are diploid (Figure 1 panel B), and iii) are viable, as shown by their ability to exclude the viability dye (7-aminoactinomycin D [7-AAD], Figure 1 panel C). Importantly, we observed that the highest number of pluripotent Oct-4^+^ VSELs reside in the population of CD133^+^Lin^−^CD45^−^UCB-derived cells [37,39]. Moreover, some of the CD133^+^Lin^−^CD45^−^ VSELs, which represent only a very small subfraction among UCB Lin^−^CD45^−^non-hematopoietic cells, may co-express other stem cell markers, including CD34, CXCR4, and SSEA-4 [37,39]. Thus, VSELs represent a very rare subpopulation of UCB stem cells among Lin^−^CD45^−^ non-hematopoietic UCB cells, which contains other stem cell types, including endothelial progenitor cells (EPCs) and mesenchymal stem cells (MSCs), and may be identified based on very small size (FSC^low^/SSC^low^) and co-expression of CD133, CD34, and, CXCR4 [37,39].

#### - In vitro and in vivo evidence that VSELs could be pluripotent stem cells

There are stringent criteria for classifying a stem cell as a PSC. In Table I are listed the most important *in vitro* and *in vivo* criteria that we expect from PSCs. These criteria were established by embryologists who are working with embryonic stem cells (ESCs) isolated from embryos or on established *in vitro*-immortalized ESC lines or induced pluripotent stem cells (iPSCs). However, some of these stringent criteria listed as *in vivo* criteria of pluripotency, such as complementation of blastocyst development and teratoma formation (Table I), are not always visible in pluripotent epiblast-derived stem cells [69,70].

**Table I:** *In vitro* and *in vivo* features of pluripotent stem cells (PSCs)

*In vitro* criteria for PSCs	VSELs
1. Undifferentiated morphology, euchromatin, and high nuclear/cytoplasm ratio 2. PSC markers (e.g., Oct-4, Nanog, and SSEA), open chromatin at the Oct-4 promoter, bivalent domains, and female PSCs reactivate the X chromosome 3. Multilineage differentiation into cells from all three germ layers (meso-, ecto-, and endoderm)	Yes Yes Yes
***In vivo* criteria for PSCs**	**VSELs**
1. Complementation of blastocyst development 2. Teratoma formation	No No

Our most recent experimental data support the conclusion that murine VSELs fulfill all the *in vitro* criteria listed in Table I. In particular, murine VSELs not only possess the primitive morphology of early developmental cells but also express typical markers for PSCs (e.g., *Oct-4, Nanog,* and *Rex-1*) [31,33,34,37,71]. Significantly, we also recently confirmed the true expression of *Oct-4* in murine VSELs (which was previously supported by the presence of an open-type chromatin in the *Oct-4* promoter) by direct analysis of its methylation state [72] and association with transcription-permissive histones [65,66]. Specifically, we observed that the *Oct-4* promoter in murine VSELs is hypomethylated, and by employing the carrier-ChIP assay, we found that the chromatin in the *Oct-4* promoter is associated with the gene transcription-promoting histone H3Ac, while its association with the transcription-restricting histone H3K9me2 is relatively low [65,66,72]. We also evaluated the epigenetic state of another core transcription factor, *Nanog*, and observed that its promoter has a higher level of methylation in VSELs (~50%). In quantitative ChIP experiments performed in parallel, we also observed that the H3Ac/H3K9me2 ratio favors transcription and supports an active state [65,66,72]. Based on these results, we conclude that murine VSELs truly express both of the embryonic transcription factors *Oct-4* and *Nanog*.

With respect to the other *in vitro* criteria of pluripotency (Table I), murine VSELs also possess bivalent domains in promoters that encode developmentally important homeobox-containing transcription factors (*Sox21, Nkx2.2, Dlx1, Lbx1h, Hlxb9, Pax5*, and *HoxA3*) [68]. Furthermore, VSELs derived from female mice reactivate the X-chromosome.

Finally, we succeeded in differentiation of VSELs *in vitro* into cells from all three germ layers, while recently published data demonstrated that murine BM-derived VSELs can be specified *in vivo* into HSCs [73], mesenchymal stem cells (MSCs) [49], and cardiomyocytes [74,75].

Our in vitro data on differentiation of human VSELs are limited at this point but so far we have shown that human UCB-derived VSELs, under appropriate culture conditions, can also be specified into the hematopoietic lineage [38].

However, taking into consideration the *in vivo* criteria expected from PSCs (Table I), murine VSELs do not complete blastocyst development and do not grow tera- tomas. This discrepancy between *in vitro* and *in vivo* criteria of PSCs for VSELs has recently been explained by epigenetic changes in expression of some paternally imprinted genes [67,72]. This phenomenon will be discussed below in the context of the developmental origin of VSELs and the mechanisms that keep them quiescent in adult tissues and prevent them from uncontrolled proliferation and teratoma formation [67,72].

### Novel molecular evidence supporting the developmental origin of VSELs

As mentioned above, gene expression studies have revealed that VSELs express several epiblast and germ line markers and we hypothesize that VSELs originate from early epiblast-derived migrating primordial germ cell (PGC)-like cells, are deposited during development in adult tissues as a source of TCSCs, and play a role in organ rejuvenation [76,77]. In support of this notion, molecular analysis of murine BM-derived VSELs has revealed that these cells express several genes that are characteristic of epiblast SCs (*Gbx2, Fgf5,* and *Nodal*) and germ line specification (*Stella, Prdm14, Fragilis, Blimp1, Nanos3, and Dnd1*) [65,66,72].

However, a main goal of the molecular analysis studies was to explain why VSELs do not fulfill the *in vivo* gold-standard criteria expected for PSCs (complementation of blastocyst development and teratoma formation in immunodeficient animals), which are seen with ESCs and iPSCs. To explain this discrepancy, we observed that VSELs, in a similar manner as late migratory primordial germ cells (PGCs), modify the methylation of imprinted genes, preventing them from uncontrolled proliferation and explaining their quiescent state in adult tissues [65,66].

It is well known that imprinted genes play a crucial role in embryogenesis, fetal growth, the totipotential state of the zygote, and the pluripotency of developmentally early stem cells [78,79]. It has been demonstrated that VSELs freshly isolated from murine BM erase the paternally methylated imprints at regulatory differentially methylated regions (DMRs) within the *Igf2-H19* and *Rasgrf1* loci; however, they also hypermethylate the maternally methylated imprints at DMRs for the Igf2 receptor (*Igf2R), Kcnq1-p57^KIP2^,* and *Peg1* loci [72].Because paternally expressed imprinted genes (*Igf2* and *Rasgrf1*) enhance embryonic growth and maternally expressed genes (*H19, p57^KIP2^,* and*Igf2R*) inhibit cell proliferation [72], the unique genomic imprinting pattern observed in VSELs demonstrates the growth-repressive influence of imprinted genes on these cells [72].

All these results suggest that epigenetic reprogramming of genomic imprinting maintains the quiescence of Oct4^+^ epiblast/germ line-derived VSELs deposited in the adult body and protects them from premature ageing and uncontrolled proliferation (*e.g.*, teratoma formation). On the other hand, reversal of this mechanism will be crucial to employing VSELs as a population of PSCs in regenerative medicine. Currently, we are testing whether downregulation of the expression of H19 enhances VSEL expansion, as has recently been demonstrated for PSCs derived by parthenogenesis [80,81].

### The role of murine VSELs in tissue rejuvenation and longevity

Interestingly, our most recent work has revealed that murine VSELs modulate by epigenetic changes the expression of imprinted genes, such as *Igf2-H19*, *RasGRF1,* and *IGF2R,* that play an important a role in insulin/insulin-like growth factors signaling (IIS), as well as a crucial role in maintaining the pool of VSELs residing in adult tissues [82,83]. Accordingly, we observed that murine BM-sorted VSELs erase the paternally methylated imprints within the DMRs for *Igf2-H19* and *RasGrf1*, while they hypermethylate the maternally methylated DMR for*Igf2R*. The epigenetic modification of imprinted loci (including *Igf2-H19, RasGRF1, and IgfR*) prevents VSELs from uncontrolled proliferation and teratoma formation. This epigenetic modification of imprinted genes explains why murine VSELs, despite expressing several markers of pluripotency (e.g., an open chromatin structure at the promoters for *Oct-4* and *Nanog*), the presence of bivalent domains at developmentally important homeobox-domain containing genes, the reactivation of the X chromosome in female VSELs, and *in vitro* differentiation into cells from all three germ layers, do not complement blastocyst development after injection into the pre-implantation blastocyst (Table I).

**Figure 2:**
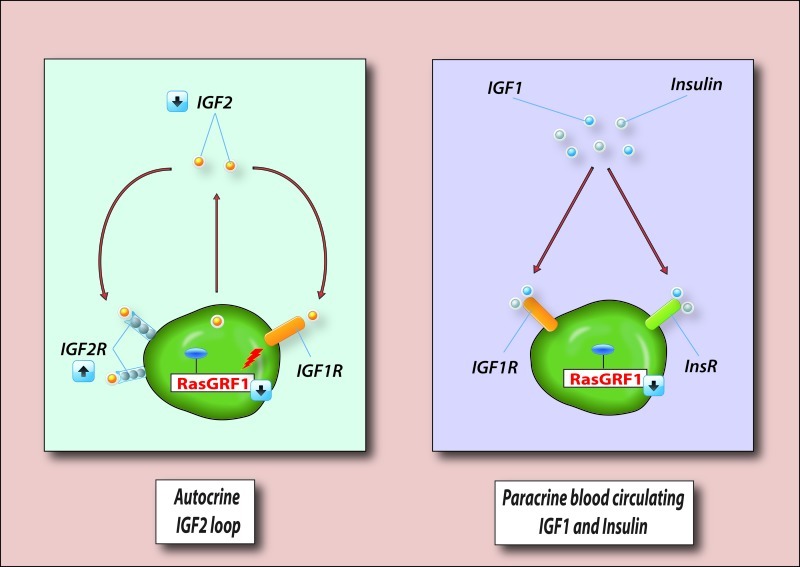
VSELs deposited in adult tissues are protected from excessive insulin/insulin-like growth factor signaling (IIS) VSELs are deposited in adult tissues as a backup population for tissue-committed stem cells (TCSCs) and are protected from IIS, which would otherwise lead to their premature depletion from adult tissues, as well as trigger uncontrolled proliferation and teratoma formation. **Left panel** - VSELs are protected from autocrine IIS by changes in expression of imprinted genes that are important in IIS. Downregulation of autocrine expression of IGF2, upregulation of IGF2R (a non-signaling receptor that binds IGF2 and prevents its binding to signaling IGF1R), and downregulation of RasGRF1 (which is involved in signal transduction from IGF2R), makes VSELs less sensitive to autocrine/paracrine IGF2 signaling. **Right panel** - Downregulation of RasGRF1, which is involved in signaling from activated IGF1R and InsR in VSELs, also plays an important role in attenuation of IIS signaling by paracrine circulating levels of IGF1 and insulin. However, in the presence of chronic elevated levels of IGF1 and insulin in blood, VSELs deposited in adult tissues may proliferate in an uncontrolled manner and become depleted much faster over time. This may contribute to the accelerated aging observed in situations with high circulating IGF1 and insulin levels (e.g., Laron dwarf mice, Ames dwarf mice, or chronic high caloric uptake). At the same time, chronic exposure to IIS may also trigger uncontrolled activation of VSELs and their malignant transformation. For reasons of simplicity, other imprinted genes not involved in IIS that negatively affect VSEL proliferation (H19 and p57^Kip2^) are not shown.

To summarize, the abovementioned changes in expression of imprinted genes in murine VSELs lead to perturbation of IIS by downregulation of i) IGF2, which is an autocrine factor involved in proliferation of VSELs, and ii) RasGRF1, which is a GTP-exchange factor (GEF) crucial for signaling from activated IGF-IR and the insulin receptor (InsR, Figure 2). In addition, hyperemethylation of DMRs on the maternal chromosome encoding IGF-2R [83] has an additional negative affect on IIS in VSELs. As mentioned above, IGF2R serves as a decoy receptor that prevents IGF-2 from binding to IGF-IR [83]. This epigenetic reprogramming of genomic imprinting negatively affects IIS signaling, maintains the quiescent state of murine VSELs, and thus protects them from premature depletion from the tissues and prevents their involvement in tumor formation.

Based on these findings and published reports that elevated IIS signaling negatively affects life span in experimental animals [84], we proposed a hypothesis that relates aging, longevity, and IIS to the abundance and function of pluripotent VSELs deposited in adult murine tissues [82,83]. We postulate that a decrease in the number of these cells due to prolonged IIS negatively affects the pools of TCSCs in various organs and has an impact on tissue rejuvenation and life span [82,83]. In support of this notion, we observed a significantly higher number of VSELs in long-living murine strains (e.g., Laron dwarfs and Ames dwarfs), whose longevity is explained by low levels of circulating IGF1 and a decrease in IIS [85]. By contrast, the number of VSELs is reduced in mice with high levels of circulating IGF1 and enhanced IIS (e.g., growth hormone-overexpressing transgenic mice) compared to normally aging littermates [85].

The influence of IIS on the pool of VSELs residing in adult tissues seems to be twofold (Figure 2). First, since IGF2 is an autocrine factor for these cells, downregulation of expression of *Igf2* and *RasGrf1* together with upregulation of *Igf2R,* which is a decoy receptor for IGF2, keeps VSELs quiescent. At the same time, these cells also have high expression of *H19* and *p57^Kip2^*, which are also regulated by imprinting, and negatively affect cell proliferation. However, with increasing age, the methylation pattern of DMRs at paternally imprinted genes in VSELs reverses and*Igf2-H19,* as well as *RasGrf1,* loci become gradually methylated, and thus VSELs become more sensitive to IIS [83], which may contribute to their age-related depletion over time.

On the other hand (Figure 2), VSELs express functional IGF-1R and InsR and are susceptible to exogenous circulating paracrine IGF1 and insulin (Ins). Therefore, a chronic increase in caloric uptake that elevates circulating levels of IGF1 and Ins may contribute over time to depletion of these cells from adult tissues, affect the generation of VSEL-derived TCSCs, and thus negatively affect life span. This explains why mice that have high levels of circulating blood plasma IGF1 and enhanced IIS display accelerated depletion of VSELs and have a shorter lifespan than age-matched littermates [85].

### Conclusions

New data from our group and other groups has provided more evidence on the existence and biological role of primitive embryonic-like stem cells in murine adult tissues and their potential role in i) tissue organ rejuvenation, ii) longevity, and iii) regeneration/repair of damaged tissues.

While murine BM-derived VSELs have been extensively characterized, we are aware that more work is needed to better characterize these small cells at the molecular level in humans. We need to determine whether human VSELs have the same molecular signature (*e.g.*, an open chromatin structure at the *Oct4* promoter, modification of somatic imprinting, and the presence of bivalent domains) as their murine counterparts. If we can confirm that a similar mechanism operates for human and murine BM-derived UCB-VSELs, perhaps a controlled modulation of the somatic imprinted state to produce proper *de novo* methylation of somatic imprinted genes on the maternal and paternal chromosomes could increase the regenerative power of these cells.

Finally, our work on murine VSELs has for the first time connected the role of caloric restriction/uptake, IIS, and the number of VSELs playing a potential role in tissue and organ rejuvenation [83,85]. An increase in chronic caloric uptake leads to an increase in IIS and may prematurely deplete VSELs from adult tissues, thus contributing to aging. By contrast, caloric restriction has an opposing beneficial effect on the tissue-residing pool of VSELs and longevity. Based on the encouraging studies on VSELs in the murine BM, further studies are needed to evaluate whether the number of VSELs in other adult murine tissues is also sensitive to IIS and correlates with life span. Similar studies have also to be performed on human VSELs.
